# New Geographies of Global Health

**DOI:** 10.4269/ajtmh.18-0190

**Published:** 2018-08

**Authors:** B. Emily Esmaili

**Affiliations:** Hubert Yeargan Center for Global Health, Duke University, Lincoln Community Health Center, Durham, North Carolina

A 10-year-old strikingly bright Afghani girl named Layla came to clinic with 7 days of unilateral cervical lymphadenopathy, persistent fevers, and a diffuse rash. She came in with her equally bright—though somewhat more humble—teenaged sister, both in hijabs. Their mother was home, looking after their gaggle of siblings. Their father was lost somewhere in Afghanistan while fleeing.

This was not in some faraway refugee camp, but rather in our clinic two miles from my North Carolina home.

Despite her piercing eyes, Layla is thin and pale at baseline—or at least has been so for the last 4 years after being forced to escape from her home. Perhaps, because of her history as a refugee, she was hospitalized for this constellation of symptoms and endured 3 days of testing, broad empiric treatment, and close observation. Mom, with limited understanding of English, could not fully understand what was being done to her daughter, nor why. Finally, after ruling out a long differential of tropical, zoonotic, and other obscure diseases, she was discharged home with presumptive treatment of standard, humdrum staphylococcal lymphadenitis.

When I saw her a few days later for follow-up and explained the reasons for hospitalization, I listened to her mother’s frustration. I wondered if the same measures would have been taken with another child who did not have the stamp of “refugee” on her chart.

Nonetheless, Layla was as bright and agreeable as before.

Global health is becoming increasingly placeless. One need not travel far to encounter tropical diseases and old-world pathologies. Perhaps, I would see more cases of leishmaniasis in the Middle East or more filariasis in Africa. However, we see plenty of foreign-born microbes and malnutrition, undiagnosed congenital syndromes, and a medley of conversion disorders in our own local refugee clinic.

Compared with clinics in shakier parts of the world, we do find more non-accidental trauma than artillery trauma and more childhood obesity than stunting, and we hear more stories about shame than terror.

Indeed, the stories we hear are enough to make one wonder why such diseases of extreme poverty and human conflict (with slightly different presentations) continue to persist here in our comfortable, safe communities. What barriers, seen and unseen, continue to confine and threaten these refugees who already have so little control and so few freedoms left?

Finding themselves in our consumer-driven, increasingly xenophobic U.S. context, refugee families now face different adversities. New mothers are isolated and depressed, leaving their toddlers with lollipops for breakfast. Teenagers wrestle with the realities of poverty and prejudice while trying to mold their futures and identities. Kindergarteners must learn to befriend children from entirely different backgrounds, perhaps while being bullied for their own.

Zaw is a 5-year-old Burmese boy with a history of a positive purified protein derivative (PPD), but that finding had been lost to follow-up. He came in for his kindergarten physical accompanied by his mother, whose warm smile was stained from years of chewing *paan* (betel nut). She carried a sling that cradled her 3-month-old drooling daughter. I was disheartened to hear that since seeing Zaw over a year ago, he continued to have nightly fevers and a relentless cough. He had also lost more weight.

After several rounds of circular Q&A, all clumsily conducted through a phone interpreter, I learned that the mom was genuinely concerned but was afraid to seek care for her son. While living in the camp in Thailand, he was diagnosed with tuberculosis (TB) and forced to live in an isolation hut for months. Neither family nor friends were allowed to visit.

She did not want him to go through that again. She simply wanted him to go to school and make the most of this new life. “*What else is there to live for*,” she said in Karen, as her baby cooed from inside her traditional plaited sling.

Refugee children and families, regardless of where on the globe they land, are branded with the stories they carry with them. Along with their luggage and rucksacks, they carry a past riddled with trauma, incessant transit, obligate poverty, unimaginable injustice, and an ultimate lack of agency. Moreover, their new homes—many of our own communities—lack the resources they need to fully integrate and, like Zaw, make the most of their new lives.

We lack multilingual, trauma-informed case managers and counselors. We terminate their health insurance if they cannot decipher the lengthy, convoluted renewal application that is all in English. We allow preconceived judgments and stigma to drive our clinical decision-making. And although global health research has started to reveal certain trends in refugee health, little attention is given to the more subtle, nuanced drivers of health and wellness among this richly diverse population. Thus, many of our newest neighbors continue to have some of the worst health indicators in our nation.

Francoise is a 14-year-old Congolese girl, born in Tanzania. Her parents sought asylum there for 22 years, awaiting their resettlement assignment. As I finished off her sports physical visit and stamped the requisite forms, her mother urged her to tell me about her “private issue.”

For the last several months, she had had abdominal pain and rectal bleeding, along with rectal prolapse so disturbing, she was afraid to tell anyone. I knew she had bouts of amoebiasis, giardiasis, and hookworm, but polyparasitemia is not uncommon in my patients—neither is debilitating fear.

After several more visits, we were able to manage the chronic constipation that was at the root of her bleeding prolapse, but only to uncover deeper, more insidious obstructions, such as the memory of rape when she walked alone to the camp latrines in her former home.

It is no longer enough to simply treat the lymphadenitis, the TB, or the parasitemia. Global health must also engage the stigmas and politics that perpetuate global and local disparities, and which imprint and disrupt these children’s lives indefinitely.

I have met children such as these in refugee camps scattered across the globe and in our North Carolina clinic. Our modest, government supported refugee clinic is stocked with textbook tropical diseases and pathologies and conditions that are harder to test and treat. Children are stunted and developmentally delayed, as most of their short lives have been spent in makeshift camps—perhaps with fragmented families, prolonged periods of food scarcity, and constant unpredictability.

And yet, I have also met many young refugees thriving in their new, cleaner, and sturdier homes. They revel in their ability to go to school every day and they take great pride in their diverse panel of compatriots. Children such as Layla, Zaw, and Francoise may carry imprints of their pasts, but their resilience and brilliance despite it all begs for a belief in second chances.

Just as the geography of global health is changing—coming closer to home and reflecting today’s dynamic sociopolitical environment—global health research and policy must shift accordingly to address the emerging, multidimensional patterns of refugee health. As national borders become increasingly porous, clinicians and researchers must also address the poverty, social and religious injustice, and human conflict that are at the root of infectious and noncommunicable tropical diseases presenting both here and abroad. We must work to dissolve the barriers dividing our clinics, communities, and patients; not just in far-off lands but also in our own neighborhoods.

Our commitment to global health is, then, a call to action; it is a call for a well-coordinated multidisciplinary effort to tackle the complex, diverse, and constantly changing pathologies common among refugees worldwide. Especially in light of today’s ever shifting flows of human migration, it is our duty—in whatever geography we find ourselves—to honor those stories entrusted to us and to protect the right to second chances, throughout the globe.

